# Metabolic Syndrome and Survival Outcomes in Endometrial Cancer

**DOI:** 10.7759/cureus.60324

**Published:** 2024-05-15

**Authors:** Alina-Gabriela Marin, Alexandru Filipescu, Radu Vladareanu, Aida Petca

**Affiliations:** 1 Obstetrics and Gynaecology, Elias Emergency University Hospital, Bucharest, ROU; 2 Obstetrics and Gynaecology, Carol Davila University of Medicine and Pharmacy, Bucharest, ROU

**Keywords:** disease-free survival, overall survival, outcome, metabolic syndrome, endometrial cancer

## Abstract

Menopause, through attributable estrogen level decline and the corresponding increase in circulating androgens, significantly elevates a woman's risk for cardiometabolic diseases, including metabolic syndrome (MetS), type 2 diabetes, and cardiovascular disease. Metabolic syndrome itself is a cluster of interconnected risk factors, and among them, central obesity is a well-established factor for the development of endometrial cancer (EC), the most common gynecologic malignancy. This research investigates the impact of metabolic syndrome on survival rates among patients with endometrial cancer. The goal is to assess whether having metabolic syndrome or its individual components influences disease-free survival (DFS), overall survival (OS), cancer-specific survival, and recurrence rates. Understanding this link is crucial for determining risk levels and could help tailor treatment approaches for better long-term outcomes in endometrial cancer care.

## Introduction and background

Endometrial cancer (EC), accounting for the majority of uterine malignancies, represents a significant health burden for women worldwide. Numerous factors are at the very core of the development of EC (in particular endometrioid subtype), including obesity, early menarche, late menopause, nulliparity, unopposed estrogen therapy, and chronic anovulation. A temporal pattern association has been described and coincides with a parallel increase in obesity and the prevalence of metabolic syndrome (MetS) observed in the general population [1,2].

MetS is a constellation of interrelated metabolic abnormalities that increase the risk of cardiovascular disease, type 2 diabetes, and certain types of cancer, including EC. Over the years, the definition of MetS suffered changes. The World Health Organization (WHO) introduced one of the earliest definitions in 1998 [3] and prioritized insulin resistance alongside two additional factors from obesity, dyslipidemia, hypertension, or microalbuminuria [4]. Due to its limitations, in 1999, the European Group for the Study of Insulin Resistance (EGIR) revised the definition. It maintained insulin resistance as a central requirement but replaced waist-to-hip ratio/body mass index (BMI) with waist circumference (WC) for obesity and omitted microalbuminuria [5]. In 2001, the definition of MetS was updated by the National Cholesterol Education Program Adult Treatment Panel III (NCEP ATP III) with an additional amendment in 2005. Accordingly, ≥3 of five criteria were required: WC, arterial hypertension, high fasting triglycerides (TG), low fasting high-density lipoprotein-cholesterol (HDL-C), and high fasting blood glucose [6,7]. In 2005, the International Diabetes Federation (IDF) introduced new criteria [8], focusing on obesity, defined by population-specific cutoff points, without necessarily requiring insulin resistance. While recognizing the importance of visceral obesity, the IDF definition has been criticized for prioritizing obesity over insulin resistance as a central factor in the pathophysiology of MetS [9].

MetS definitions were comparatively evaluated to ensure consistency. Thus, a Chinese study evaluated the prevalence of MetS using both IDF and revised ATP III criteria in over 15,800 adults. While the prevalence differed (16.5% for IDF and 23.3% for ATP III), there was a high level of agreement (93.2%) between the two approaches [10].

There is growing evidence indicating a relationship between metabolic health, obesity, and the risk of developing neoplasia. MetS conceals a group of cardiovascular risk factors including obesity, which has, as underlying mechanisms, insulin resistance and hormonal imbalances. This connection is particularly evident in the increased risk of EC, especially in the obesity-related subtype. However, a more nuanced picture emerges when considering metabolically healthy obese (MHO) and metabolically unhealthy normal weight (MUNW) individuals. MHO individuals, despite obesity, do not display the metabolic impairments of MetS, and research suggests a lower risk of cardiovascular disease and mortality when compared to MUNW counterparts. Intriguingly, MHO has been associated with an enhanced risk of specific cancers, including EC [11-13].

## Review

Menopause and a rising tide of metabolic syndrome

Existing data suggest a high prevalence of MetS after menopause, ranging from 31% to 55%. This represents a significant increase compared to premenopausal women [14,15]. The decrease in estradiol (E2) levels seen during menopause may contribute to the metabolic dysregulation and increased adiposity often seen in this population, suggesting that menopause is a possible independent risk factor for MetS [16-20]. Supporting this notion, a large Chinese meta-analysis identified statistically significant differences (p < 0.01) in all MetS components (WC, BMI, TG, low-density lipoprotein-cholesterol (LDL-C), systolic blood pressure (SBP), diastolic blood pressure (DBP), fasting glucose, and insulin) except HDL-C (p = 0.72) between premenopausal and postmenopausal women [21].

Likewise, a vast US meta-analysis encompassing over 95,000 postmenopausal women reported a pooled prevalence of MetS exceeding 37% (95% confidence interval (CI): 35.00%-39.31%), associating significant heterogeneity between studies (ranging from 13.6% to 46%). This disparity is likely attributed to the use of different diagnostic criteria for MetS. Furthermore, this US meta-analysis provided compelling evidence for a substantially higher risk of individual MetS components in postmenopausal women in comparison to premenopausal women. This included higher odds ratios (OR) for increased WC (OR: 2.75, 95% CI: 1.80-4.21), high blood pressure (OR: 3.95, 95% CI: 2.01-7.78), elevated fasting glycemia (OR: 3.51, 95% CI: 2.11-5.83), low HDL-C (OR: 1.45, 95% CI: 1.03-2.03), and high triglyceride levels (OR: 3.20, 95% CI: 2.37-4.31) [22].

Time since menopause and tailored risk assessment

The impact of menopause on MetS goes beyond a simple pre- versus post-distribution [23,24]. Research from Korea, analyzing over 1,000 women, suggests a link between the elapsed time since menopause and the specific components of MetS a woman might experience. Postmenopausal women within five years of menopause are at higher risk of abdominal obesity and hyperglycemia. Women between five and nine years postmenopausal have the strongest association with arterial hypertension, while those between 10 and 14 years postmenopausal have an elevated risk of hypertriglyceridemia [25].

Surgical menopause and increased MetS risk

Studies show a significantly higher prevalence of metabolic disorders following surgical menopause compared to natural menopause. This association is linked to the sudden decline in estrogen levels caused by surgery, which can lead to endothelial dysfunction, dyslipidemia [1,26-29], and increased risk of cardiovascular events [30-34]. Research suggests a 1.5-fold to 9.7-fold increased risk of MetS in women who undergo surgical menopause compared to natural menopause [1,35-39].

Furthermore, research suggests distinct glycemia profiles according to the type of menopause. Women with a history of surgical menopause had significantly higher two-hour postprandial and postprandial plasma glucose concentrations compared to those who transitioned naturally [40-44].

Tailoring hormone replacement therapy (HRT) for metabolic benefits

Studies suggest that the type of estrogen in HRT influences its impact on MetS [45,46]. E2-based HRT improves triglyceride levels and diastolic blood pressure, while conjugated equine estrogen (CEE) affects LDL-C and HDL-C levels [21]. Further supporting the potential of HRT, a large US meta-analysis observed a 6.8% reduction in abdominal fat (95% CI: -11.8% to -1.9%), a 12.9% reduction in homeostatic model assessment-insulin resistance (HOMA-IR) (95% CI: -17.1% to -8.6%), and a 30% reduction in the relative risk of developing new diabetes (95% CI: 0.6 to 0.9) among women without diabetes. Women with pre-existing diabetes also experienced improved blood sugar control (95% CI: -18.0% to -5.1%) and lowering of HOMA-IR (95% CI: -51.7% to -19.8%). Additionally, HRT lowered cardiovascular risk factors such as LDL/HDL ratio (95% CI: -18.0% to -13.5%) and lipoprotein(a) (Lp(a)) (95% CI: -32.9% to -17.1%) and modulated inflammatory markers [47].

Endometrial cancer and the metabolic shadow: a complex interplay

EC is strongly associated with a cluster of metabolic disorders known as the "metabolic triad": obesity, type 2 diabetes mellitus (DM), and arterial hypertension [48]. Studies show a 2.45-fold to 3.5-fold increased risk of EC in overweight and obese with high blood pressure compared to controls. These observations reinforce EC as one of the gynecological malignancies significantly associated with metabolic disorders [49].

Delving deeper, US research investigated the link between MetS and individual factors contributing to the development of EC. The study investigated over 24,000 women and explored the impact of WC within the MetS definition established by the NCEP ATP III. It was observed that the inclusion of WC in the MetS definition showed a link to a twofold hazard ratio (HR) for EC (HR: 2.20, 95% CI: 1.61-3.02), a risk that disappeared when WC was removed from the MetS definition. This suggests that central adiposity plays a significant role in the EC-MetS connection. Furthermore, the study revealed that the association of hyperglycemia, dyslipidemia, and arterial hypertension within the MetS criteria independently increased cancer risk by an HR of 1.94 (95% CI: 1.09-3.46), regardless of WC status [50].

Large studies in China and Korea also confirm this association between MetS and EC risk. The Korean study further found that both overweight and obesity independently increase EC risk. When compared with normal-weight women, overweight women presented a 36% higher risk (HR: 1.36, 95% CI: 1.28-1.45), while obese women presented almost twice the risk (HR: 1.92, 95% CI: 1.82-2.04). Specifically, high WC conferred the highest risk of EC (HR: 1.42, 95% CI: 1.34-1.50 for MetS; HR: 1.49, 95% CI: 1.42-1.57 for high WC) [11,51].

Endometrial cancer outcome

EC has a relatively low estimated lifetime risk of 0.96%, with a corresponding mortality risk of 0.23%, translating into a favorable mortality-incidence ratio of 0.24, considerably lower than other gynecologic malignancies such as breast cancer (0.32), ovarian cancer (0.63), and cervical cancer (0.55) [52]. Also, a substantial proportion (75%) of ECs are diagnosed in the early stages (International Federation of Gynecology and Obstetrics (FIGO) I or II), leading to high five-year overall survival (OS) rates ranging between 74% and 91%. As for patients diagnosed with advanced forms of the disease (FIGO III and IV), the five-year OS rates decrease to 57%-66% and 20%-26%, respectively. Likewise, disease-free survival (DFS) rates reflect a stage-dependent pattern, with an estimated rate of 90% in patients without lymph node involvement, 60%-70% with pelvic lymph node involvement, and 30%-40% with para-aortic lymph node involvement [53].

Apart from the FIGO stage, a constellation of clinicopathological factors, such as age at diagnosis, pre-existing comorbidities, tumor grade and diameter, lymphovascular space involvement (LVSI), American Society of Anesthesiologists (ASA) score, and occurrence of complications within the first 30 days postoperatively, has a significant impact on survival in EC patients [49].

A recent Chinese review explored mortality patterns and underlying causes of death in patients diagnosed with EC. Mortality was highest within the first five years post-diagnosis (42.1%), with a gradual decline thereafter (20.5% at 1-5 years, 21.6% at 5-10 years, and 15.8% at >10 years). Nearly half (42.9%) of deceased patients succumbed directly to EC. However, a substantial portion died from other causes: 15.6% from secondary malignancies and 41.5% from non-cancer-related illnesses. Patients with localized disease, negative lymph node involvement (N0 stage), well-differentiated tumors (grade I/II), and endometrioid carcinoma histology exhibited a higher propensity for non-cancer mortality. Heart disease constituted the leading non-cancer cause of death (12.8%), followed by cerebrovascular disease (3.3%), diabetes mellitus (2.3%), and Alzheimer's disease (2.2%). Interestingly, EC survivors displayed a consistently elevated risk of death from diabetes compared to the general population [54].

MetS also gained increasing consideration as a potential contributor to EC survival. Therefore, a Canadian case-control study explored, over an average duration of 14.2 years, the prognostic significance of MetS and WC in a population of 540 EC survivors, 60.2% of whom had MetS. Women with EC and MetS associated with a worse OS (HR: 1.98, 95% CI: 1.07-3.67), but no statistically significant endometrial cancer-specific survival (HR: 1.80, 95% CI: 0.75-4.33). Also, a high WC (≥88 cm) emerged as an independent prognostic indicator for worse overall survival (OS) (HR: 2.12, 95% CI: 1.18-3.80). However, no statistically significant associations were observed between high WC and disease-free survival (HR: 1.66, 95% CI: 0.98-2.79), endometrial cancer-specific survival (HR: 2.14, 95% CI: 0.73-6.31), or recurrence (HR: 1.21, 95% CI: 0.58-2.52) [55].

A Chinese retrospective study evaluated the prognostic impact of MetS on 385 women with EC. Univariate analysis revealed a statistically significant association between MetS and poorer OS (p = 0.001). The assessment also showed a significant positive correlation between MetS and the clinicopathological features associated with more aggressive EC: tumor size (p = 0.035), stage (p = 0.021), histological grade (p = 0.022), vascular invasion (p = 0.044), and lymphatic metastasis (p = 0.014). Furthermore, among 129 patients with coexisting MetS and EC, a univariate analysis of OS identified the following significant prognostic factors (all p < 0.05): advanced tumor stage, presence of vascular invasion, tumor size exceeding 2 cm, lymphatic metastasis, and high cancer antigen 19-9 (CA19-9) level (>37 U/mL) [56].

A Chinese retrospective analysis evaluated data from 506 patients diagnosed with EC, 30.2% of whom were diagnosed with MetS. They observed a positive correlation between MetS and aggressive tumor features, including higher histological grade, advanced stage, lymph node metastasis (LNM), lymphovascular space invasion (LVSI), and myometrial invasion (MI). Regarding survival rates, univariate analysis revealed a significant association between MetS and both OS and recurrence-free survival (RFS) in EC patients. Patients with MetS had an HR of 2.14 for death (p = 0.032) and an HR of 1.80 for recurrence (p = 0.045) compared to the non-MetS group. Kaplan-Meier analysis corroborated these findings, demonstrating a statistically significant decrease in both OS and RFS for patients with MetS. Notably, the presence of three or more MetS components further worsened patient outcomes compared to those with zero or one to two components (p < 0.05). However, after adjusting for age, histological type, tumor grade, and stage in a multivariate model, only high-density lipoprotein cholesterol (HDL-C) emerged as a significant independent predictor of mortality (HR: 2.2; p = 0.034). The association between MetS and survival did not retain statistical significance in this model. Receiver operating characteristic (ROC) curve analysis demonstrated that combining factors such as HDL-C, tumor grade, and stage yielded superior predictive power for one-, three-, and five-year survival rates compared to traditional staging or grading alone [57].

A UK meta-analysis evaluating the effect of diabetes on survival outcomes in over 55,000 EC patients observed that patients with diabetes exhibited a demonstrably higher HR of 1.15 (95% CI: 1.00-1.32, I² = 62%) for cancer-specific mortality compared to the diabetes-free group. This translates to an approximately 15% increased risk of death specifically attributable to EC in diabetic patients. Furthermore, diabetic patients associated a significantly higher HR of 1.23 (95% CI: 1.02-1.47, I² = 0%) for disease recurrence or progression. Additionally, a statistically significant association was observed between diabetes and a poorer OS rate (HR: 1.42, 95% CI: 1.31-1.54, I² = 46%) [58].

However, it is important to acknowledge some conflicting data. A recent German retrospective bicentric study did not find a statistically significant difference in survival rates between patients with and without MetS. It comprised 415 patients with a median age of 64 years who were followed up for a median duration of 43 months. Assessment of the association between MetS and oncological parameters did not reveal statistically significant differences in either progression-free survival (PFS) or OS between groups. The median PFS for the MetS group was 36 months compared to 40 months for the non-MetS group (HR: 1.49, 95% CI: 0.79-2.80; p = 0.210), in contrast to obese patients who had a demonstrably shorter PFS compared to the non-obese group (34.5 versus 44.0 months; p = 0.029). Similarly, no significant disparity in survival was observed, with a median OS of 38 months for the MetS group and 43 months for the non-MetS group (HR: 1.66, 95% CI: 0.97-2.87; p = 0.063) [59].

Future directions

It is crucial to delve deeper into the biological pathways linking menopause to the development of MetS and also to develop a more nuanced approach to risk stratification. Future research should focus on identifying modifiable factors (lifestyle habits and environmental exposures) and non-modifiable factors (genetic predisposition and pre-existing medical conditions) that influence individual susceptibility to MetS after menopause. Rigorous clinical trials are needed to assess the effectiveness of different interventions in preventing or mitigating MetS in postmenopausal women. These interventions could include lifestyle changes (diet, exercise), pharmacological approaches (targeted therapies), and the potential benefits of HRT.

## Conclusions

In conclusion, our study provides strong evidence for the association between MetS and EC, particularly the increased risk observed in postmenopausal women compared to premenopausal controls. While the prognostic role of MetS in EC has not been definitively established, our findings strongly support obesity as an independent predictor of poorer survival outcomes. This underscores the potential of lifestyle interventions in the prevention and management of EC. In addition, the observed heterogeneity in MetS prevalence underscores the critical need for standardized diagnostic criteria in future research to comprehensively elucidate the complex relationship between MetS and EC. By further delineating the synergistic interactions between metabolic dysfunction and endometrial carcinogenesis, we hope to catalyze significant advances in disease prevention and improve patient prognosis, ultimately reducing the burden of EC on women's health.
